# The impact of SARS-CoV-2 on respiratory syndromic and sentinel surveillance in Israel, 2020: a new perspective on established systems

**DOI:** 10.2807/1560-7917.ES.2022.27.16.2100457

**Published:** 2022-04-21

**Authors:** Aharona Glatman-Freedman, Lea Gur-Arie, Hanna Sefty, Zalman Kaufman, Michal Bromberg, Rita Dichtiar, Alina Rosenberg, Rakefet Pando, Ital Nemet, Limor Kliker2,, Ella Mendelson, Lital Keinan-Boker, Neta S Zuckerman, Michal Mandelboim, on behalf of The Israeli Respiratory Viruses Surveillance Network (IRVSN)

**Affiliations:** 1 The Israel Center for Disease Control, Israel Ministry of Health, Tel Hashomer, Ramat Gan, Israel; 2 Department of Epidemiology and Preventive Medicine, School of Public Health, Faculty of Medicine, Tel Aviv University, Tel Aviv, Israel; 3 The Central Virology Laboratory, Israel Ministry of Health, Tel Hashomer, Ramat Gan, Israel; 4 School of Public Health, University of Haifa, Israel; 5 The Israeli Respiratory Viruses Surveillance Network (IRVSN) members are listed under Acknowledgements

**Keywords:** SARS-CoV-2, Sentinel surveillance, Syndromic surveillance

## Abstract

**Background:**

The COVID-19 pandemic presented new challenges for the existing respiratory surveillance systems, and adaptations were implemented. Systematic assessment of the syndromic and sentinel surveillance platforms during the pandemic is essential for understanding the value of each platform in the context of an emerging pathogen with rapid global spread.

**Aim:**

We aimed to evaluate systematically the performance of various respiratory syndromic surveillance platforms and the sentinel surveillance system in Israel from 1 January to 31 December 2020.

**Methods:**

We compared the 2020 syndromic surveillance trends to those of the previous 3 years, using Poisson regression adjusted for overdispersion. To assess the performance of the sentinel clinic system as compared with the national SARS-CoV-2 repository, a cubic spline with 7 knots and 95% confidence intervals were applied to the sentinel network's weekly percentage of positive SARS-CoV-2 cases.

**Results:**

Syndromic surveillance trends changed substantially during 2020, with a statistically significant reduction in the rates of visits to physicians and emergency departments to below previous years' levels. Morbidity patterns of the syndromic surveillance platforms were inconsistent with the progress of the pandemic, while the sentinel surveillance platform was found to reflect the national circulation of SARS-CoV-2 in the population.

**Conclusion:**

Our findings reveal the robustness of the sentinel clinics platform for the surveillance of the main respiratory viruses during the pandemic and possibly beyond. The robustness of the sentinel clinics platform during 2020 supports its use in locations with insufficient resources for widespread testing of respiratory viruses.

## Introduction

The severe acute respiratory syndrome coronavirus 2 (SARS-CoV-2) which emerged in Wuhan, China, at the end of 2019 [1], spread rapidly throughout the world [2], causing the coronavirus disease (COVID-19) pandemic and challenging healthcare systems of high-, middle- and low-income countries [3]. In parallel to the management of COVID-19 patients, a great need for population-based information emerged. The need for such information was particularly evident in the early stages of the pandemic given the global shortage of testing kits, reagents and the long turn-around time for laboratory processing [4]. One of the key recommendations of the World Health Organization (WHO) was to ensure that the existing surveillance of respiratory disease was maintained [5].

A national influenza surveillance system was established in Israel in 1997. It includes several surveillance platforms based on data from outpatient clinics, emergency departments and a sentinel clinic network consisting of primary care clinics. The sentinel clinics are situated in all seven districts of Israel, and are run by internists, family physicians and paediatricians. During the 2019/20 influenza season, 32 sentinel clinics were part of this network.

The first case of COVID-19 was identified in Israel on 20 February 2020 (week 8). While during the following 3 weeks, the sole modality for following the outbreak consisted of epidemiological investigations and contact tracing, the rapidly growing number of new patients that followed the carnival holiday of Purim on 10–11 March 2020 (week 12) highlighted the need for population-based information. Therefore, the long-standing influenza surveillance system was adapted in week 13, 2020 into a COVID-19 surveillance system [6] and from week 40 onwards, into a combined influenza and COVID-19 (FLURONA) surveillance system.

The objective of this study was to evaluate the performance and outcome of our surveillance system under the new circumstances of the year 2020.

## Methods

### Surveillance period

We evaluated the surveillance system from week 1 (ending 4 January) to week 53 (ending 31 December) in 2020.

### Syndromic surveillance

Several platforms were used to monitor morbidity during 2020. Those consisted of both community and emergency department platforms.

#### Patient visits to primary care physicians

To monitor morbidity in the community, we followed patient's visits to primary care physicians of the second largest healthcare maintenance organisation (HMO) in Israel, which provides healthcare services to 25% of the Israeli population. Specifically, we followed weekly consultations for upper respiratory infection (URI), influenza-like illness (ILI) and pneumonia. The diagnoses were recorded by physicians at the conclusion of each visit and were not based on specific case definitions. Return visits (visits with the same diagnosis) within 28 days were excluded. Visits were expressed as rates per 10,000 HMO members, and per 10,000 members of specific age groups. We evaluated the age groups < 2, 2–5, 6–11, 12–18, 19–64 and ≥ 65 years. During 2020, visits to a physician for all causes were added to the syndromic surveillance. Both face-to-face and virtual visits in 2020 were included in the surveillance and the analysis.

#### Emergency department visits

We evaluated emergency department (ED) visits to eight medical centres for pneumonia. These eight medical centres belong to a single HMO, constitute 30% of the general medical centres in Israel and are located in the north, centre and south of the country. During 2020, ED visits for all causes were added to the syndromic surveillance. The ED visits for all causes were expressed as crude numbers. The ED visits for pneumonia were expressed as crude numbers and as percentage of the ED visits for all causes. The diagnoses were recorded by physicians at the conclusion of each visit.

### Sentinel surveillance

Sentinel surveillance was performed in sentinel clinics spread throughout Israel. During epidemiological weeks 1–11, the clinics followed the long-standing ILI case definition, which has been routinely used for sentinel surveillance in Israel, and for which samples are routinely tested for influenza virus and for respiratory syncytial virus (RSV). This case definition includes the following symptoms: a temperature of ≥ 37.8 °C with at least one of the following symptoms: rhinorrhoea, cough, sore throat and muscle ache. Medical teams were provided the option to include other symptoms and/or signs that appeared important. Starting in week 13 of 2020, the clinics were requested to obtain nasopharyngeal samples from patients who fulfilled the case definition of a suspected COVID-19 case (see below), but did not have a history of exposure to a laboratory-confirmed COVID-19 patient or history of returning from foreign travel in the 14 days before illness onset. Symptomatic patients with a history of contact or foreign travel were eligible for SARS-CoV-2 testing by non-sentinel procedure based on guidelines from the Israel Ministry of Health (MOH). The case definition was based on an MOH directive issued before the detection of the first COVID-19 case in Israel [7] and included: fever > 38 °C or at least one of the following symptoms: cough, difficulty breathing or any other acute respiratory symptom. Starting in week 40 of 2020, the case definition underwent another change in which the temperature cut-off was determined at ≥ 37.8 °C, similar to influenza season surveillance. The other components of the case definition remained the same as those implemented in week 13. Supplementary Table S1 outlines the case definitions used by sentinel clinics, as well as the viruses tested for throughout 2020. Convenience sampling has been implemented in all sentinel clinics during influenza seasons and throughout the year 2020. The samples were transported to the MOH Central Virology Laboratory in cooling conditions. Starting in week 13, the samples were transported to the laboratory twice a week (as compared with once a week during influenza surveillance seasons). In the early stages of the pandemic, sentinel clinic patients who were quarantined were sampled in their homes by emergency services medical staff [6]. Those samples were tested by other laboratories that were approved by MOH to perform SARS-CoV-2 PCR testing.

Starting in week 13 of 2020, the Israel Center for Disease Control (ICDC) provided the sentinel clinics with personal protective equipment (PPE) for use during sampling. The PPE included surgical masks, visors, gowns and gloves [6].

### National repository of SARS-CoV-2 PCR test results

A national SARS-CoV-2 PCR test repository was established at the beginning of the COVID-19 pandemic in Israel to capture all SARS-CoV-2 PCR test results from all laboratories approved by the MOH to perform the test. The repository included result of each SARS-CoV-2 test, date of testing and the date when the result was available. Non-sentinel PCR testing was based on symptoms (e.g fever > 38 °C and/or acute respiratory symptoms) and/or exposure to SARS-CoV-2-positive contacts and/or foreign travel, as well as belonging to high-risk populations. Thus, PCR testing outside the sentinel clinics was not limited to individuals who presented with symptoms, and the repository included tests results from both symptomatic and non-symptomatic individuals. The number and rate of weekly SARS-CoV-2 cases in Israel were calculated based on the repository data. To calculate the number of cases, only the first positive PCR test of each individual was considered. Additional positive PCR tests for the same individual were excluded from analysis.

### Laboratory methods

Viral nucleic acid was extracted from sentinel samples using the MagNA PURE 96 system (Roche, Mannheim, Germany). Real-time RT-PCR reactions were conducted using primers targeting the SARS-CoV-2 envelope (E) and nucleoprotein (N) genes based on a previously described protocol [8]. All RT-PCR reactions were conducted using the Ambion Ag-Path Master Mix (Life Technologies, Austin, Texas, United States (US)) and TaqMan Chemistry on ABI 7500 instruments.

### Statistical analysis

Rates of weekly patient visits per 10,000 HMO members were calculated for all ages and for specific age groups. We calculated percentages of weekly visits to internal medicine and paediatric ED and of weekly positive SARS-CoV-2 samples. Rates of national weekly SARS-CoV-2 cases were calculated based on the national SARS-CoV-2 repository data.

Patients visit rates to outpatient clinics for URI, ILI, pneumonia and all diagnoses in 2020 were compared with each of the years 2017 to 2019 by using Poisson regression adjusted for overdispersion.

To compare the sentinel network weekly percentage of SARS-CoV-2-positive cases to the weekly percentage of the national SARS-CoV-2-positive cases, a cubic spline with seven knots with 95% confidence intervals (CI) was applied to the sentinel network weekly percentage of positive SARS-CoV-2 cases.

Statistical analysis was performed with statistical software programmes R version 3.5.2 (R foundation, Vienna, Austria) and SAS Enterprise Guide 7.1 (SAS Institute Inc., Cary, North Carolina, US).

## Results

The evolution of the SARS-CoV-2 pandemic in Israel during 2020 is demonstrated in Supplementary Figure S1. Specifically, the number of weekly new and cumulative SARS-CoV-2 cases, based on national SARS-CoV-2 PCR testing are shown as well as with key events in the timeline. The weekly number of SARS-CoV-2 patients was based on the national SARS-CoV-2 repository data.

### Syndromic surveillance

#### Outpatient visits

##### Upper respiratory infection 

**Figure 1:**
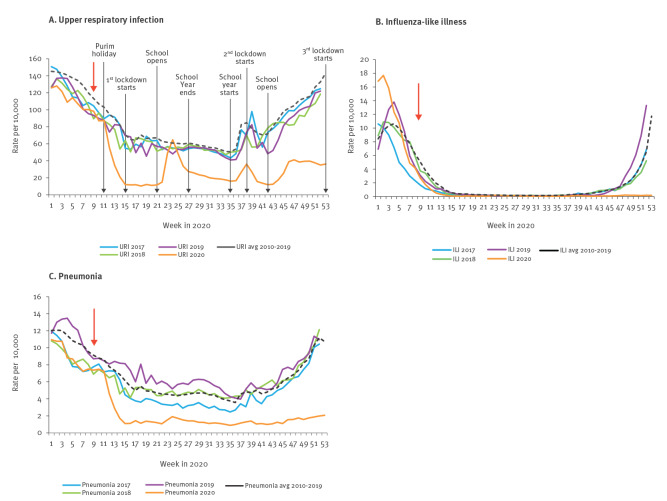
Rates of community physicians' visits for three respiratory conditions used in syndromic surveillance, Israel, 2017-2020

**Figure 2:**
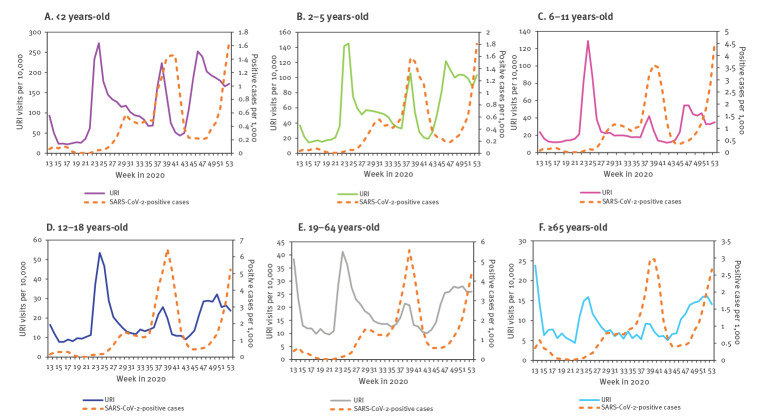
Rates of weekly physicians' visits for upper respiratory infections and rates of national SARS-CoV-2-positive cases, by age group, Israel, 2020

SARS-CoV-2: severe acute respiratory syndrome coronavirus 2; URI: upper respiratory infection.

Weekly URI visit rates are shown in Figure 1, panel A. Starting in week 3 of 2020, the rate of visits to physicians declined. Until week 11, this decline was similar to that of previous seasons. However, starting in week 12, the decline became steeper and URI reached rates below those observed in previous seasons. The rates remained substantially below previous seasons' rates for most weeks of the evaluation period. The difference between rates in 2020 vs each of the years 2017 to 2019 was statistically significant (p value < 0.01). A rise in the rate of URI visits occurred three times during the evaluation period: during weeks 23–26, weeks 37–39 and weeks 41–55. These URI increases occurred primarily among children younger than 11 years (the weekly rate of visits to primary care physicians for upper respiratory infection (URI) by age for 2020 is provided in Supplementary Figure S2). The URI increases started 1–2 weeks after school openings (Figure 1, panel A). The URI morbidity pattern of the second rise in URI visit rate was in line with the rates in the national SARS-CoV-2 case repository for all age groups (Figure 2). The first and the third rise in URI rates were inconsistent with the rise in the rates of national SARS-CoV-2 cases for all age groups (Figure 2).

Avg: multi-year average; ILI: influenza-like illness; URI: upper respiratory infection.

Red vertical arrows denote the week in which the first COVID-19 case was diagnosed in Israel. Black vertical arrows denote relevant events during 2020.

##### Influenza-like illness

Weekly ILI visit rates are shown in Figure 1, panel B. Starting in week 3 of 2020, the rate of physicians' visits for ILI declined. This decline was similar to that in the previous winter season, and minimal ILI activity was observed in weeks 13 and 14, representing the end of the 2019/20 influenza season. During the rest of 2020, ILI activity remained at inter-influenza season levels. The difference between rates in 2020 and each of the years 2017 to 2019 for weeks 40 to 52 was statistically significant (p value < 0.05 compared with years 2017 and 2018 and < 0.01 compared with 2019).

##### Pneumonia

Weekly rates of physicians' visit for pneumonia are shown in Figure 1, panel C. From week 1 until week 11 in 2020, we observed a gradual decline in the rate these visits, similar to that of previous years. Starting in week 12 of 2020, a sharp decline occurred. The rates remained substantially below previous seasons' rates for the rest of 2020. The difference between the rates in 2020 and the rates of each of the years 2017 to 2019 was statistically significant (p value < 0.0001).

##### All diagnoses

**Figure 3:**
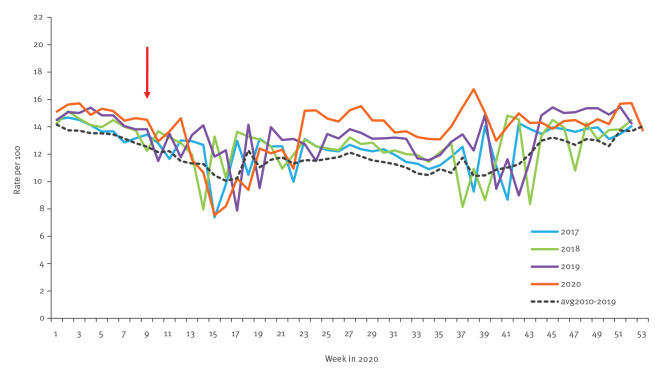
Weekly rate of visits to primary care physicians for all diagnoses used in syndromic surveillance, by year and multi-year average, Israel, 2017–2020

The rate of physician's visits for any diagnosis was maintained close to or above the multi-year average level for most weeks of 2020 (Figure 3). Between weeks 23 and 38, the 2020 rate exceeded those of the previous 3 years and that of the multi-year average (Figure 3). The difference in weekly rates between the year 2020 and each of the years 2017 and 2018 were statistically significant (p value < 0.01). However, the difference between 2020 and 2019 was not statistically significant.

Avg: multi-year average.

The red vertical arrow denotes the week in which the first COVID-19 case was diagnosed in Israel.

#### Emergency department visits

**Figure 4:**
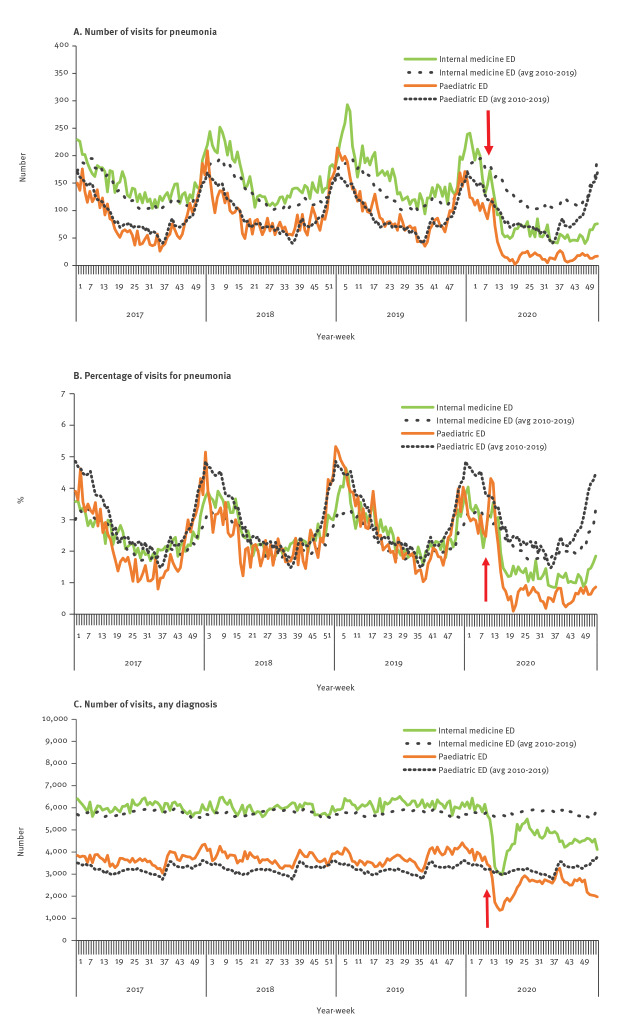
Weekly visits to emergency departments, Israel, 2017–2020

ED weekly surveillance for pneumonia and for all diagnoses is shown is Figure 4.

Avg: multi-year average; ED: emergency department.

Red vertical arrows denote the week in which the first COVID-19 case was diagnosed in Israel.

##### Pneumonia

The number and the percentage of ED visits for pneumonia decreased gradually during the first 8 weeks of 2020, similar to previous years. Starting in week 14 of 2020, the number and rate of ED visits fell to below seasonal levels. This decline occurred in both paediatric and internal medicine EDs (Figure 4A and B). The difference between the rates in 2020 and each of the years 2017 to 2019 was statistically significant (p value < 0.01) for both paediatric and internal medicine EDs.

##### All diagnoses

The number of ED visits for all diagnoses decreased gradually during the first 9 weeks of 2020. Starting in week 13 of 2020, the number and rate of ED visits fell drastically (Figure 4C). Although the rates increased by week 25, they remained below seasonal levels for the rest of 2020 in the internal medicine ED and for most weeks in the paediatric ED (Figure 4C). The difference between rates in 2020 and each of the years 2017 to 2019 was statistically significant (p value < 0.01) for both paediatric and internal medicine EDs.

### Sentinel surveillance

#### SARS-CoV-2 circulation

Sentinel surveillance for SARS-CoV-2 started in week 13 of 2020. It included 36 to 48 clinics and collected 3,180 samples through week 53. Of those, 147 (4.6%) were positive for SARS-CoV-2.

**Figure 5:**
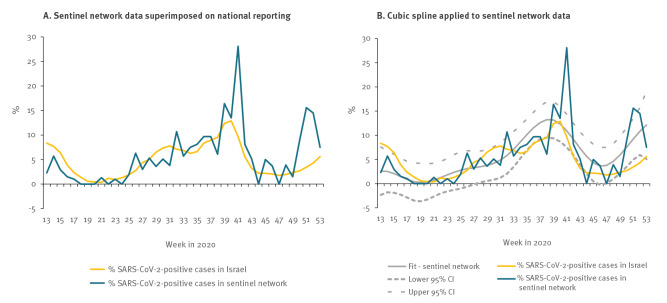
Percent positive SARS-CoV-2 cases in the sentinel network superimposed on the percent of the national Israeli SARS-CoV-2-positive cases, with Cubic spline applied to the sentinel network percent SARS-CoV-2-positive cases, Israel, 2020

CI: confidence interval; SARS-CoV-2: severe acute respiratory syndrome coronavirus 2.

Figure 5A presents the percentage of weekly positive cases from the sentinel network superimposed on the percentage of the SARS-CoV-2 cases collected in the national repository. Figure 5B presents the application of cubic spline with 95% CI to the sentinel clinic curve. The trends in the percentage of weekly positive cases in the national SARS-CoV-2 curve data points were included within the 95% CI of the cubic spline of the sentinel network curve (Figure 5B).

#### Circulation of influenza and respiratory syncytial viruses

During the first 11 weeks of 2020, influenza and RSV detections in sentinel clinics were consistent with the seasonal pattern. Influenza and RSV sentinel surveillance for the 2020/21 season, which started in week 40 of 2020, did not detect influenza or RSV in samples obtained at sentinel clinics through week 53 of 2020. The number of weekly influenza- and RSV-positive samples from sentinel clinics during the 2020/21, 2019/20, 2018/19 and 2017/18 seasons are provided in Supplementary Figures S3 and S4. 

## Discussion

The COVID-19 pandemic presented several challenges for the Israeli surveillance system. The decline in patient visits for respiratory diagnoses to physician's offices and EDs modified our ability to understand the seasonal trends of respiratory illness during 2020. This decline was most probably due to public concern about visiting medical facilities and contracting COVID-19 and to the implementation of non-pharmaceutical intervention and physical distancing measures.

A decline in physician's visits for respiratory illness was also observed during 2020 in the United Kingdom (UK) [9], except for ILI rates which exceeded the UK baseline in April 2020 [9]. A surge in physicians' visits for ILI was also observed in France and the US in March 2020 [10,11]. These increases in physicians' visits for ILI paralleled high rates of COVID-19 cases and consultations [9-11]. In Israel, ILI rates did not increase during surges of COVID-19 cases. A decline in ED visits was described in reports from several countries, suggesting similar effect of the pandemic on ED visits globally [12-14].

It is important to note that the decline in patient visits for respiratory illness started before any substantial increase in COVID-19 cases in Israel. This early decline was probably due to public health warnings regarding the new coronavirus, and the implementation of public health measures before the first COVID-19 cases in Israel, both affecting the population's behaviour. As a result, patient visits to primary clinics and EDs for respiratory illness could not contribute to early warning of COVID-19 cases in Israel.

It is also interesting to note that while ED visits for all diagnoses declined to below seasonal levels, community physician visits for all diagnoses did not decline. The latter is probably due to the implementation of virtual physician visits by Israeli HMOs in the early stages of the pandemic.

Despite the overall lower rate of URI physicians' visits, school openings were associated with increases in physicians' visits for URI. However, since these did not consistently parallel the rises in national SARS-CoV2 case rates, other pathogens may have been involved. In this regard, previous studies demonstrated that school openings were associated with increase in respiratory infection and school closures were associated with their decline [15,16].

The availability of the national SARS-CoV-2 testing system provided a unique (and first, to our knowledge) opportunity to evaluate the robustness of the sentinel surveillance system during the first year of the pandemic which included three waves of transmission in Israel. A previous publication from Israel [6] assessed only the first pandemic wave (which had the least number of patients as compared with the other two waves) and lacked the perspective of a full year's follow-up and the evaluation of multiple syndromic surveillance platforms. The sentinel surveillance of SARS-CoV-2 followed a trend similar to that of the national SARS-CoV-2 testing system. While the sentinel SARS-CoV-2 surveillance system was less sensitive during weeks with very low SARS-CoV-2 circulation, it demonstrated the ability to serve as a marker of SARS-CoV-2 circulation during most weeks of the pandemic. Although the case definition for sentinel surveillance was modified twice during 2020, these changes did not appear to affect the similarity in trend between the two testing systems. Furthermore, although the PCR testing outside the sentinel clinics was not limited to individuals who presented with symptoms, and the repository included tests results from both symptomatic and non-symptomatic individuals, both systems followed a similar trend. It is worth noting that in order to compensate for the reduced number of patient clinic visits for respiratory diagnoses, we recruited additional clinics to our sentinel clinic network, reaching a maximum of 48 clinics (compared with 32 clinics in the network in the influenza season that preceded the pandemic).

The rates of SARS-CoV-2-positive children aged 6–11 and 12–18 years registered in the Israel national repository were equivalent to those in older age groups. A recent systematic review and meta-analysis showed that children transmit the virus to other children at lower rates than to adults [17]. Another study found that there was no difference in the transmission of SARS-CoV-2 in children compared with adults in households [18]. Based on recent data, the average number of births per woman in Israel is 3.0, while it is about half of that in many European countries and the US [19]. Thus, one infected adult in Israel, can potentially infect more children (in a family or other close contact setting) as compared with European countries and the US. 

Whole genome next generation sequencing performed on SARS-CoV-2-positive sentinel samples (Supplement pages 6–9 and Supplementary Figure S5 include the methodology, the results and a phylogenetic tree) provided additional support for the robustness of our SARS-CoV-2 sentinel surveillance. The largest proportion of samples from sentinel clinics belonged to clade 20C, which is predominantly a North American clade, followed by clades 20B and 20A which originated in Europe [20]. These findings are consistent with recent data demonstrating that travellers from the US made the most substantial contribution to the spread of SARS-CoV-2 in Israel, followed by travellers from Europe [21].

The lack of viruses belonging to the 19A and 19B clades, which were predominant in Asia during the initial period of the SARS-CoV-2 outbreak, is consistent with their global decrease, as clade 20 became the dominant strain worldwide, around March 2020 (https://nextstrain.org/ncov/global?dmax=2020-12-3). MOH restrictions on travel from South-East Asia to Israel, implemented in the early stages of the pandemic, may have contributed to their absence in Israel.

None of the sequenced sentinel samples during 2020 were associated with any global variant of concern (VOC). Most sentinel samples were collected before the introduction and spread of VOCs in Israel. The first emerging VOCs, B.1.1.7 and B.1. 351 were first detected in September and October of 2020, respectively [22]. However, they were first identified in Israel only in December 2020 [23,24]. 

The lack of influenza or RSV detections between weeks 40 and 53 in 2020 are consistent with findings in the northern hemisphere during the same weeks [25,26], and those of the southern hemisphere 2020 autumn/winter surveillance [27,28]. These findings could be a result of non-pharmaceutical interventions, physical distancing measures and reduction in international travel.

## Conclusion

Our work demonstrates modified patterns of the syndromic surveillance platforms during 2020, presenting a challenge for surveillance. On the other hand, the sentinel surveillance component provided information that was consistent with SARS-CoV-2 morbidity and genomic sequencing patterns in Israel. Our findings highlight the importance of surveillance systems for respiratory pathogens in general and SARS-CoV-2 in particular. They further support the need to create and maintain robust surveillance systems, which can be expanded in the context of public health emergencies in order to monitor the spread of newly emerging pathogens. Robust sentinel surveillance is of particular importance in places and circumstances lacking sufficient capacity for a widespread testing capacity for respiratory pathogens.

## Supplementary Data

335000 Supplement
