# Supplementary material for: The impact of SARS-CoV-2 on respiratory syndromic and sentinel surveillance in Israel, 2020: a new perspective on established systems
Source: Euro Surveill. 2022 Apr 21;27(16):2100457. doi: 10.2807/1560-7917.ES.2022.27.16.2100457 (PMC9027148; doi:10.2807/1560-7917.ES.2022.27.16.2100457)
Supplement: Supplement [file 21-00457_GLATMAN_Supplement.pdf]

Supplementary materials for the manuscript:

The impact of SARS-CoV-2 on respiratory syndromic and sentinel surveillance in Israel, 2020: A new perspective on established systems

Aharona Glatman-Freedman, Lea Gur-Arie, Hanna Sefty, Zalman Kaufman, Michal Bromberg, Rita Dichtiar, Alina Rosenberg, Rakefet Pando, Ital Nemet, Limor Kliker, Ella Mendelson, Lital Keinan-Boker, Neta S. Zuckerman, Michal Mandelboim on behalf of The Israeli Respiratory Viruses Surveillance Network (IRVSN)

This supplementary material is hosted by *Eurosurveillance* as supporting information alongside the article "*The impact of SARS-CoV-2 on respiratory syndromic and sentinel surveillance in Israel, 2020: a new perspective on established systems*" on behalf of the authors who remain responsible for the accuracy and appropriateness of the content. The same standards for ethics, copyright, attributions and permissions as for the article apply. Supplements are not edited by *Eurosurveillance* and the journal is not responsible for the maintenance of any links or email addresses provided therein.

Contents

Page

Table S1 - Case definitions used by sentinel clinics in Israel during 2020……………………………………………….2

Figure S1 - Number of weekly new and cumulative SARS-CoV-2 cases in Israel, 2020 (based on the national SARS-COV-2 PCR tests repository)……3

Figure S2 - Weekly rate of visits to primary care physicians due to upper respiratory infection (URI) by age and year, 2017-2020……………………….4

Figure S3 - Number of weekly influenza-positive samples from sentinel clinics, by season ……………………………….5

Figure S4 - Number of weekly RSV-positive samples from sentinel clinics, by season ……………………………………….5

Whole genome next generation sequencing NGS for SARS-CoV-2 ……………………………………………………….6

List S1. GISAID accession numbers of submitted sentinel clinic samples……………………………………………………8

Figure S5 - A phylogenetic tree of SARS-CoV-2 viruses obtained at sentinel clinics and analyzed by whole genome next generation sequencing…..9

REFERENCES……10

Table S1. Case definitions used by sentinel clinics in Israel during 2020

Dates	Case definition for sentinel clinic Network	Not included in case definition of sentinel clinics	Viruses tested	Surveillance
**Weeks 1-11, 2020**	Fever ≥37.8^{0}C and at least one of the following symptoms: cough, difficulty breathing, coryza, sore throat, Myalgia, another relevant symptom	NA	Influenza<br>RSV	Pre-pandemic respiratory viruses sentinel surveillance of 2019-2020
**Week 12, 2020**	NA	NA	NA	Preparation for pandemic surveillance
**Weeks 13-40, 2020**	Fever >38^{0}C or at least one of the following symptoms: cough, difficulty breathing, or any other acute respiratory symptom.	Symptomatic patients with a history of contact or foreign travel (these patients were evaluated as part of epidemiological investigations)	SARS-CoV-2	Pandemic surveillance
**Weeks 40-53, 2020**	Fever ≥37.8^{0}C and/or at least one of the following symptoms: cough, difficulty breathing, coryza, sore throat, Myalgia, another relevant symptom	Symptomatic patients with a history of contact or foreign travel (these patients were evaluated as part of epidemiological investigations)	SARS-CoV-2<br>Influenza<br>RSV	Pandemic<br>and respiratory viruses surveillance

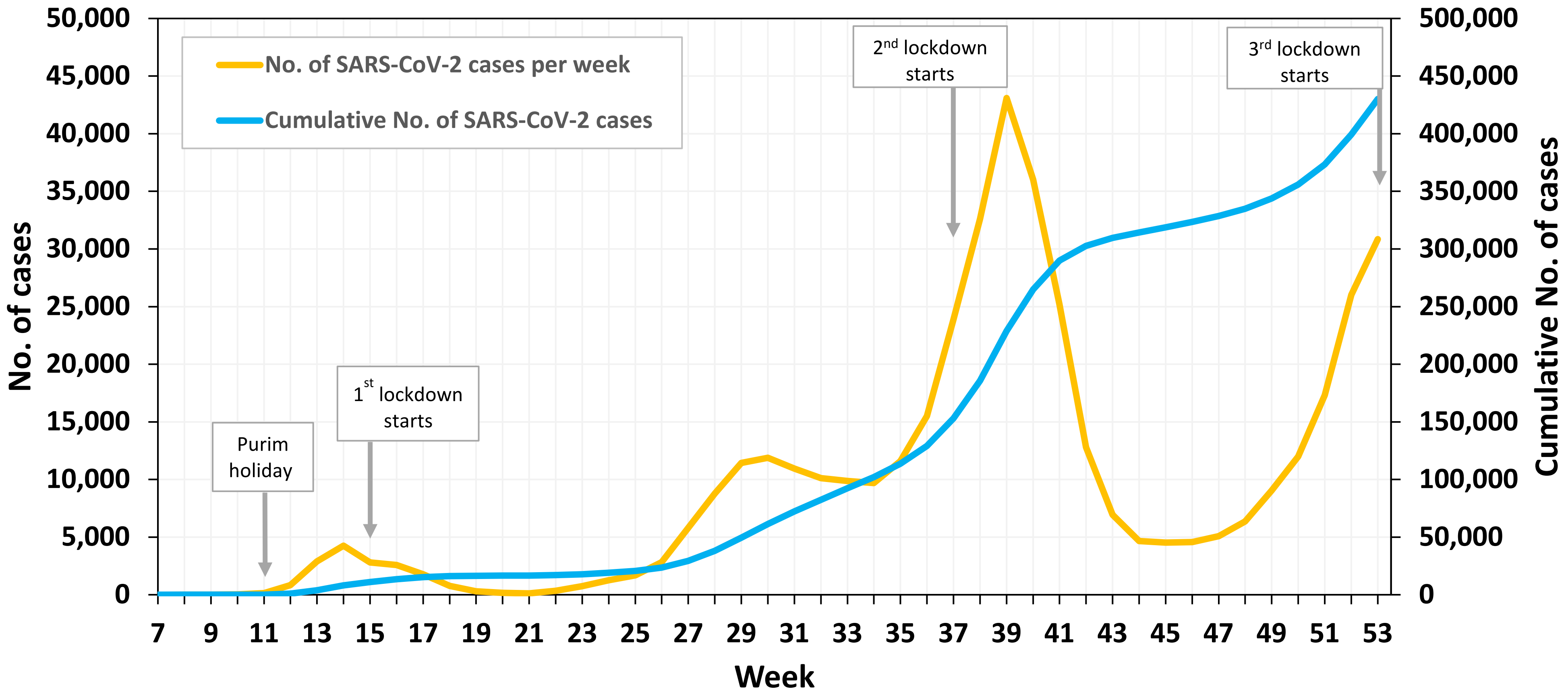

Figure S1. Number of weekly new and cumulative SARS-CoV-2 cases in Israel, 2020 (based on the national SARS-COV-2 PCR tests repository).

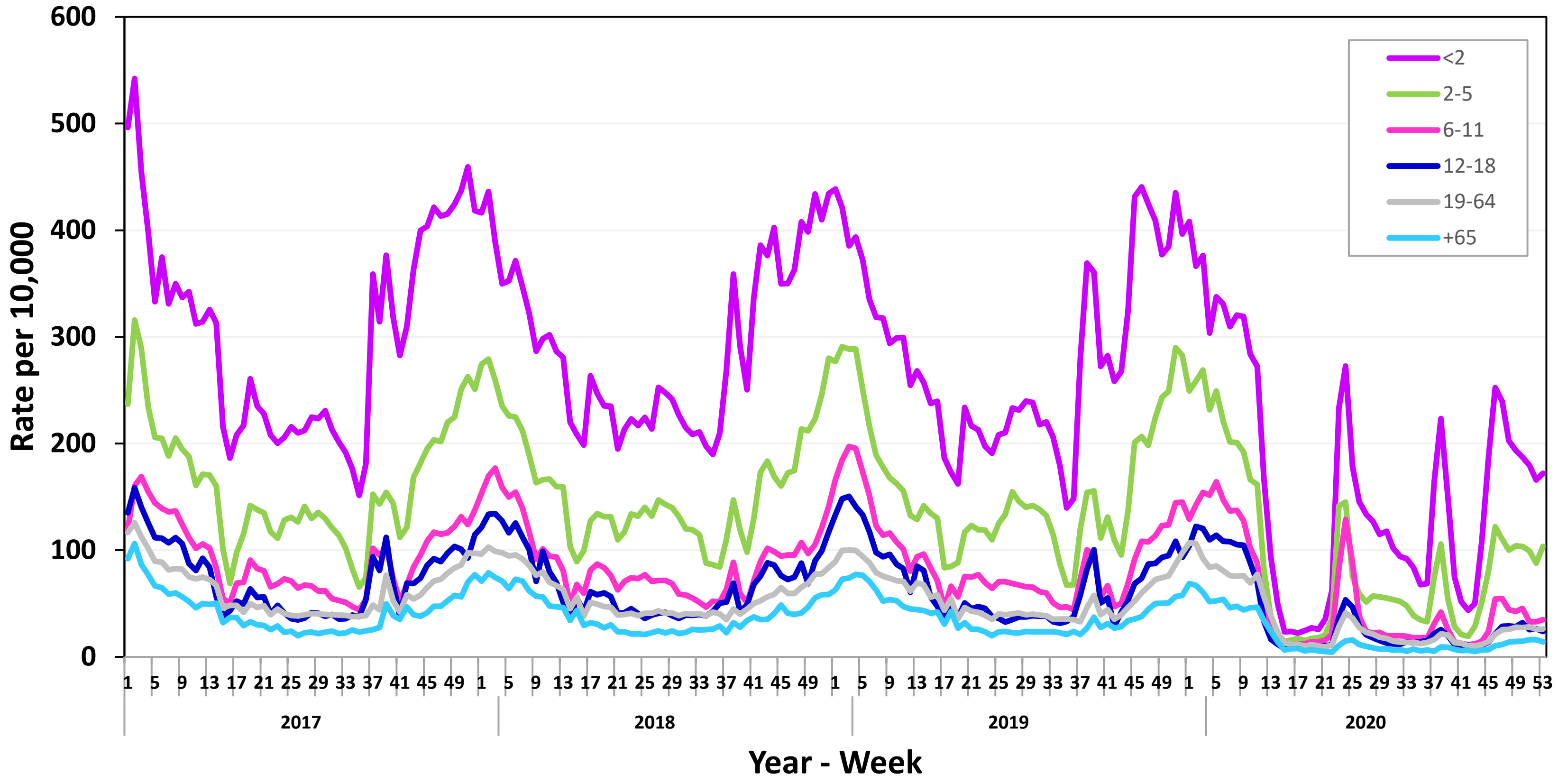

Figure S2. Weekly rate of visits to primary care physicians due to upper respiratory infection (URI) by age and year, 2017-2020.

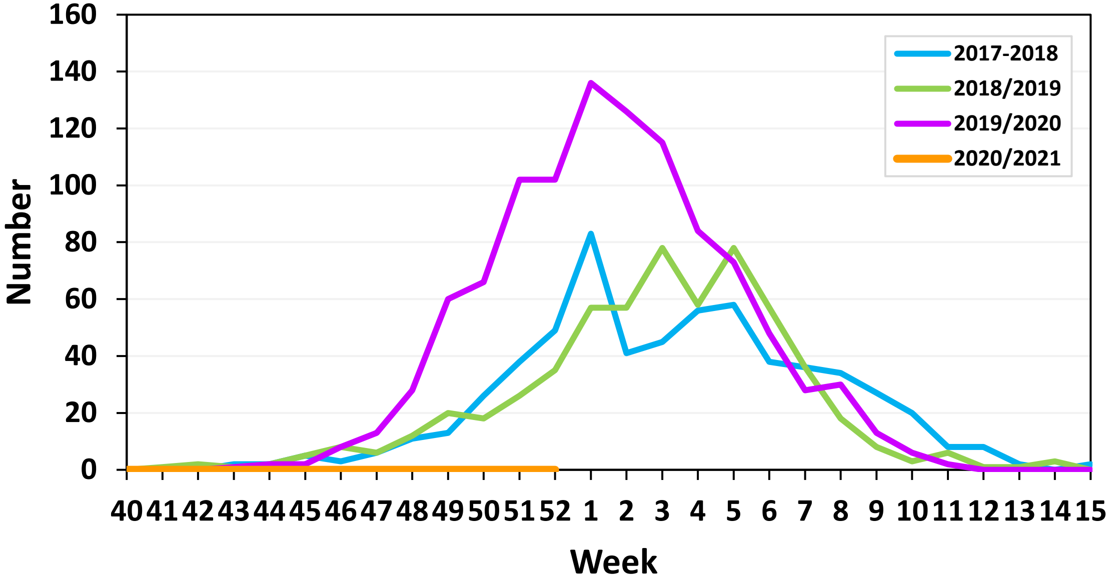

Figure S3. Number of weekly influenza-positive samples from sentinel clinics, by season.

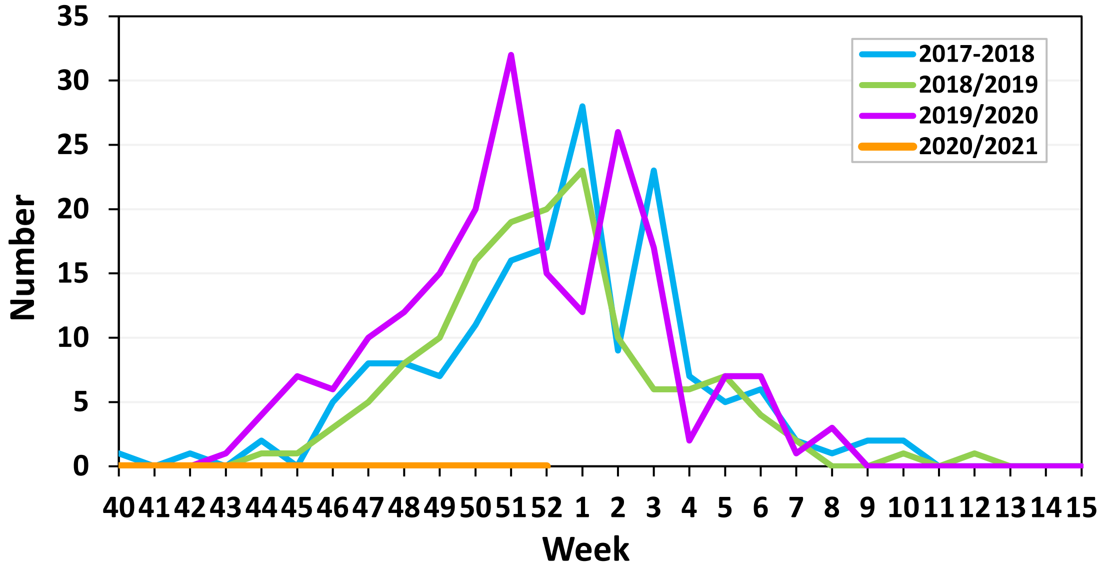

Figure S4. Number of weekly RSV-positive samples from sentinel clinics, by season.

Whole genome next generation sequencing NGS for SARS-CoV-2 in Israel

Amplification of SARS-CoV-2 from clinical samples

RNA from extractions underwent reverse transcription to single strand cDNA using SuperScript IV (ThermoFisher Scientific, Waltham, MA, USA) according to manufacturer's instructions. SARS-CoV-2 specific primers that were designed to capture SARS-CoV-2 whole genome (version 3 - total 218 primers, divided to two primer pools designed by ARTIC Network (https://artic.network/ncov-2019) (1), were used to produce double strand cDNA and amplify it by PCR using Q5 Hot Start DNA Polymerase (New England Biolabs, Inc., MA, USA). Each sample went through two PCR reactions using primer pool 1 or 2 and 5X Q5 reaction buffer, 19mM dNTPs and nuclease-free water. The resultant DNA was combined and quantified using Qubit dsDNA BR Assay kit (ThermoFisher Scientific, Waltham, MA, USA) according to manufacturer's instructions; 1ng of amplicon DNA in 5μl per sample was reserved for library preparation.

Library preparation and sequencing for SARS-CoV-2

Libraries were prepared with NexteraXT library preparation kit and NexteraXT index kit V2 according to manufacturer's instructions (Illumina, San Diego, CA, USA). Libraries were purified using AMPure XP magnetic beads (Beckman Coulter, Brea, CA, USA). Library concentrations were measured by Qubit dsDNA HS Assay kit (Thermo Fisher Scientific, Waltham, MA, USA). Library validation and fragment mean sizes were determined by the TapeStation 4200 via DNA HS D1000 kit (Agilent, Santa Clara, CA, USA). Fragment mean size was ~400 bp, as anticipated. Library fragment mean size

and molar concentration were calculated and each library was diluted to 4 nM. Libraries were pooled, denatured, diluted to 10pM and then sequenced on MiSeq using V3 2X300 bp run kit (Illumina Inc., San Diego, MA, USA).

Bioinformatics analysis for SARS-CoV-2

Fastq files were subjected to quality control via FastQC (https://www.bioinformatics.babraham.ac.uk/projects/fastqc/) and MultiQC; low-quality sequences were filtered using trimmomatic (2). SARS-CoV-2 reference genome was obtained from the national center for biotechnology information (NCBI) (NC_045512.2) and indexed with Burrows-Wheeler aligner (BWA) (3). Fastq files were mapped to the indexed reference genome by BWA mem (3). SAMtools suite (4) was utilized to filter unmapped reads, sort and index bam files. A consensus sequence was constructed for each sample with SAMtools mpileup and bcf tools (5) and then converted to a fasta file using seqtk (https://github.com/lh3/seqtk). Ns were inserted in position having depth of sequencing lower than 5 using a custom python code. Using the Augur pipeline (6), resulting consensus sequences were aligned to the SARS-CoV-2 reference genome (NC_045512.2) using MAFFT (7). A time-resolved phylogenetic tree was generated using IQ-Tree (8) and TreeTime (9) under the GTR substitution model and visualized using auspice (6). Clade nomenclature was obtained from Nextstrain (10). All sequences were submitted to GISAID. The accession numbers are detailed in List 1S.

Molecular characterization of SARS-CoV-2

Of the 147 SARS-CoV-2-positive samples collected by the sentinel network clinics, 96 (65%) underwent whole genomes sequencing, with all samples having >95% genome coverage and average sequencing depth of ~2000. A phylogenetic tree constructed via Nextstrain augur pipeline (6) depicts the

different SARS-CoV-2 lineages for the surveyed samples (Figure 6). All samples were associated with clade 20, with 55 (57.3%) samples associated with clades 20C, 24 (25%) associated with clade 20B, and 17 (17.7%) associated with clade 20A. None of the samples were associated with the origin clade 19. None of the sequenced samples were associated with any global variant of concern (VOC).

List S1. GISAID accession numbers of submitted sentinel clinic samples.

EPI_ISL_1023271, EPI_ISL_1073570, EPI_ISL_1073624, EPI_ISL_1209623, EPI_ISL_1209980, EPI_ISL_1210189, EPI_ISL_1210194, EPI_ISL_1210199, EPI_ISL_1210206, EPI_ISL_1210231, EPI_ISL_1210261, EPI_ISL_1210278, EPI_ISL_1210294, EPI_ISL_1240643-EPI_ISL_1240679, EPI_ISL_1259208, EPI_ISL_1259210-EPI_ISL_1259212, EPI_ISL_1259263-EPI_ISL_1259264, EPI_ISL_1259279-EPI_ISL_1259280, EPI_ISL_575332-EPI_ISL_575333, EPI_ISL_804175, EPI_ISL_814095, EPI_ISL_814155, EPI_ISL_814164, EPI_ISL_814185, EPI_ISL_814223, EPI_ISL_889025-EPI_ISL_889027, EPI_ISL_889047, EPI_ISL_889062, EPI_ISL_889076-EPI_ISL_889083, EPI_ISL_889086-EPI_ISL_889096, EPI_ISL_889111-EPI_ISL_889123, EPI_ISL_889226, EPI_ISL_889228-EPI_ISL_889230, EPI_ISL_944287, EPI_ISL_944312, EPI_ISL_944339, EPI_ISL_944484-EPI_ISL_944485.

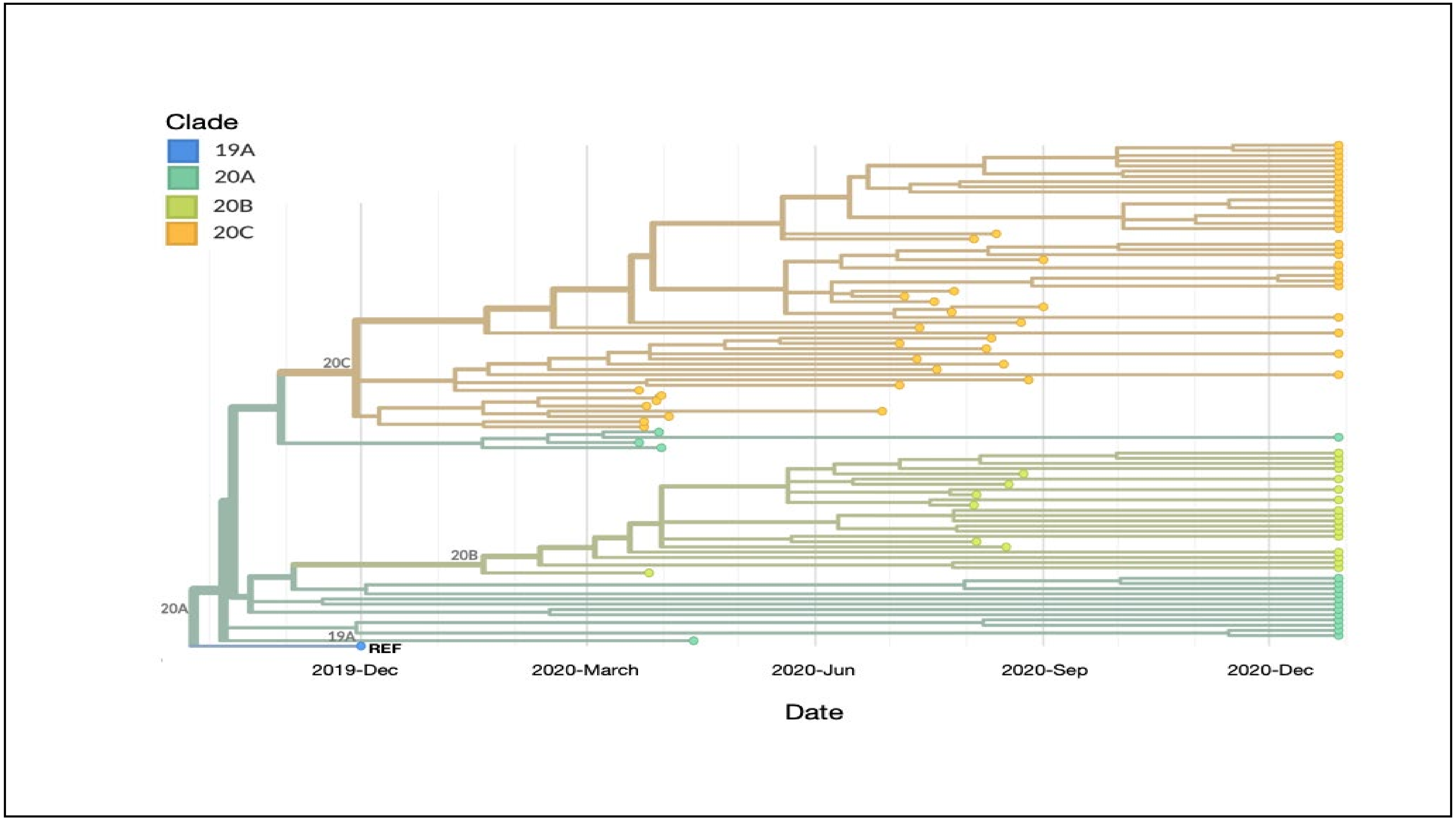

Figure S5. A phylogenetic tree of SARS-CoV-2 viruses obtained at sentinel clinics and analyzed by whole genome next generation sequencing.

REFERENCES

1. Ewels P, Magnusson M, Lundin S, Käller M. MultiQC: summarize analysis results for multiple tools and samples in a single report. Bioinformatics (Oxford, England). 2016;32(19):3047-8.
2. Bolger AM, Lohse M, Usadel B. Trimmomatic: a flexible trimmer for Illumina sequence data. Bioinformatics (Oxford, England). 2014;30(15):2114-20.
3. Li H, Durbin R. Fast and accurate short read alignment with Burrows-Wheeler transform. Bioinformatics (Oxford, England). 2009;25(14):1754-60.
4. Li H, Handsaker B, Wysoker A, Fennell T, Ruan J, Homer N, et al. The Sequence Alignment/Map format and SAMtools. Bioinformatics (Oxford, England). 2009;25(16):2078-9.
5. Danecek P, McCarthy SA. BCFtools/csq: haplotype-aware variant consequences. Bioinformatics (Oxford, England). 2017;33(13):2037-9.
6. Hadfield J, Megill C, Bell SM, Huddleston J, Potter B, Callender C, et al. Nextstrain: real-time tracking of pathogen evolution. Bioinformatics (Oxford, England). 2018;34(23):4121-3.
7. Katoh K, Standley DM. MAFFT multiple sequence alignment software version 7: improvements in performance and usability. Molecular biology and evolution. 2013;30(4):772-80.
8. Nguyen LT, Schmidt HA, von Haeseler A, Minh BQ. IQ-TREE: a fast and effective stochastic algorithm for estimating maximum-likelihood phylogenies. Molecular biology and evolution. 2015;32(1):268-74.
9. Sagulenko P, Puller V, Neher RA. TreeTime: Maximum-likelihood phylodynamic analysis. 2018;4(1):vex042.
10. Hodcroft EB, Hadfield J, Neher RA, Bedford T. Year-letter Genetic Clade Naming for SARS-CoV-2 on Nextstrain.org 2020. Available from: https://nextstrain.org/blog/2020-06-02-SARSCoV2-clade-naming.
